# Circulation of the Cultivable Symbiont *Serratia symbiotica* in Aphids Is Mediated by Plants

**DOI:** 10.3389/fmicb.2019.00764

**Published:** 2019-04-15

**Authors:** Inès Pons, François Renoz, Christine Noël, Thierry Hance

**Affiliations:** Earth and Life Institute, Biodiversity Research Centre, Université catholique de Louvain, Louvain-la-Neuve, Belgium

**Keywords:** horizontal transfers, environmental acquisition, plant-mediated transmission, insect-microbe associations, trophic networks

## Abstract

Symbiosis is a common phenomenon in nature that substantially affects organismal ecology and evolution. Fundamental questions regarding how mutualistic associations arise and evolve in nature remain, however, poorly studied. The aphid-*Serratia symbiotica* bacterium interaction represents a valuable model to study mechanisms shaping these symbiotic interspecific interactions. *S. symbiotica* strains capable of living independently of aphid hosts have recently been isolated. These strains probably resulted from horizontal transfers and could be an evolutionary link to an intra-organismal symbiosis. In this context, we used the tripartite interaction between the aphid *Aphis fabae*, a cultivable *S. symbiotica* bacterium, and the host plant *Vicia faba* to evaluate the bacterium ability to circulate in this system, exploring its environmental acquisition by aphids and horizontal transmission between aphids via the host plant. Using molecular analyses and fluorescence techniques, we showed that the cultivable *S. symbiotica* can enter the plants and induce new bacterial infections in aphids feeding on these new infected plants. Remarkably, we also found that the bacterium can have positive effects on the host plant, mainly at the root level. Furthermore, our results demonstrated that cultivable *S. symbiotica* can be horizontally transferred from infected to uninfected aphids sharing the same plant, providing first direct evidence that plants can mediate horizontal transmission of certain strains of this symbiont species. These findings highlight the importance of considering symbiotic associations in complex systems where microorganisms can circulate between different compartments. Our study can thus have major implications for understanding the multifaceted interactions between microbes, insects and plants.

## Introduction

Symbiotic associations are widespread in nature and can take different forms. Some symbiotic microorganisms are harmful (parasites), while others can be beneficial for their hosts (mutualists) (Kikuchi and Fukatsu, 2014). These interactions are, however, not fixed and can evolve along a symbiotic continuum depending on the environment in which they live (Parmentier and Michel, 2013; Sánchez et al., 2016). Symbioses occur among a wide range of organisms, with insects comprising the largest group (Kikuchi et al., 2007). In general, insects that feed on a nutritionally limited diet, such as plant sap or blood, have to deal with the lack of some essential nutrients (Frago et al., 2012). The association with mutualistic obligate symbionts that complement the host insect with these essential nutrients has allowed these organisms to access otherwise unusable resources and to evolve as complex entities (Buchner, 1965; Baumann, 2005; Dale and Moran, 2006; Gündüz and Douglas, 2009; Snyder et al., 2010; Vigneron et al., 2014). Symbiotic bacteria can thus play a crucial role in the evolution and ecology of their hosts by bringing new beneficial biological properties, while at the same time benefiting from food, means of dispersal or multiplication (Duron and Hurst, 2013; Oliver et al., 2014). Explaining how these associations are formed and spread through insect populations remains one of the most fundamental questions within the field of symbiosis research.

To ensure the fixation and persistence of such symbiotic associations, various sophisticated mechanisms of transmission may take place (Bright and Bulgheresi, 2010; Salem et al., 2015). Vertical transfer of symbionts from parent to offspring is one important way by which beneficial associations can be maintained (Salem et al., 2015). Transmission processes may differ between obligate and facultative symbionts (Kikuchi et al., 2007). Obligate associations such as tsetse flies-*Wigglesworthia glossinidia* and aphids-*Buchnera aphidicola* show host-symbiont phylogenetic congruence, suggesting evolutionary ancient and stable associations are maintained by a strict vertical transmission (Moran et al., 1993; Clark et al., 2000; Wernegreen, 2002). In contrast, in facultative associations, symbiont phylogeny rarely reflects host phylogeny, suggesting that the symbiont can also experience occasional horizontal transfers (Sandström et al., 2001; Clayton et al., 2012; Gehrer and Vorburger, 2012). Stable symbiotic associations without vertical transmission are, however, also found in nature as shown by associations between bean bugs and *Burkholderia* or between squids and *Vibrio* (Schultze and Kondorosi, 1998; Nyholm and McFall-Ngai, 2004; Kikuchi et al., 2007; Caspi-Fluger et al., 2012; Gonella et al., 2015). Life in symbiosis is not required for these microorganisms and a free-living population may serve as the inoculum for the emergence of symbiosis (Bright and Bulgheresi, 2010; McFrederick et al., 2017).

Aphids (Hemiptera: Aphididae) represent a valuable model to study the mechanisms involved in symbiont transmissions. Aphids typically harbor an obligate symbiont that provides essential amino acids that are deficient in plant phloem on which aphids feed (Douglas, 1998), but aphids can also harbor a wide variety of facultative symbionts (Oliver et al., 2010), known to be associated with benefits to aphid hosts (Montllor et al., 2002; Oliver et al., 2003; Scarborough et al., 2005; Burke et al., 2009; Tsuchida et al., 2010; Simon et al., 2011). *Serratia symbiotica* is one of the most frequent facultative symbionts of aphids (Henry et al., 2015). This species in particular is of interest, because it includes different strains associated with very distinct biological characteristics. In the *Lachninae* subfamily, *S. symbiotica* supplements the metabolic abilities of *Buchnera aphidicola* for tryptophan synthesis and has been depicted as a co-obligate partner (Gosalbes et al., 2008; Lamelas et al., 2011), whereas in the aphid *Acyrthosiphon pisum, S. symbiotica* is a facultative endosymbiont generally associated with heat stress tolerance and parasitoid resistance for its host (Oliver et al., 2003; Burke et al., 2009). In addition, some *S. symbiotica* strains have been isolated from aphids in the genus *Aphis* and cultivated freely on a pure artificial medium (Sabri et al., 2011; Grigorescu et al., 2017). These strains represent the only symbiotic bacteria of aphids that has successfully been reared in pure culture (Sabri et al., 2011), suggesting that they potentially can live in a simple environment independently of an insect host. The absence of an interdependence with aphid hosts, as well as genomic features, suggest that cultivable *S. symbiotica* strains are involved in the early stages of symbiosis with aphids, having diverged little from their free-living counterparts (Manzano-Marín et al., 2016; Renoz et al., 2017). A new study further revealed that these cultivable *S. symbiotica* strains would reside naturally in the aphid guts (Renoz et al., 2018), contrary to what is generally observed with this endosymbiont. Taken together, these results suggest that these *S. symbiotica* strains may potentially serve as an environmental reservoir for symbiotic bacteria acquisition. It is therefore critical to understand how these cultivable *S. symbiotica* strains circulate in the environment to decipher their acquisition route and their possible horizontal transmission.

Infections through the environment could provide new beneficial traits to aphid hosts creating a pathway for the emergence of new symbiotic associations. Although no study has shown the presence of *S. symbiotica* bacteria freely in the environment, it is well known that bacteria of the *Serratia* genus are capable to grow in a variety of environments, such as animals, soil and plants, including phytopathogens, as well as mutualists improving the health and development of their host plants (Petersen and Tisa, 2013). In a recent study, we showed that the cultivable *S. symbiotica* (CWBI-2.3^T^) can be either beneficial or parasitic for its aphid hosts depending on the ecological context and we also presented evidence that this cultivable *S. symbiotica* is extracellularly transmitted to future generations, potentially via contamination with honeydew (Pons et al., 2019). Nevertheless, the ability of the cultivable strain to colonize plants as well as, its biological effects on infected plants are currently unknown. Moreover, given that cultivable *S. symbiotica* is potentially located in the aphid gut, the plant may well be another source of transfer. Indeed, the role of plants in insect symbionts transmission is attracting more and more attention (Caspi-Fluger et al., 2012; Gonella et al., 2015; Li et al., 2018). Nonetheless, although phylogenetic analyses suggest some horizontal transfers or repeated infections from an exogenous source (Sandström et al., 2001; Darby and Douglas, 2003; Russell et al., 2003), there is little direct evidence that host plants can mediate horizontal transfers of aphid symbionts. The cultivable *S. symbiotica* are thus of great interest to study horizontal transmissions of aphid symbionts.

To gain insight into the circulation of the cultivable *S. symbiotica* bacterium (CWBI-2.3^T^) between the aphid *Aphis fabae* and the host plants *Vicia faba*, we experimentally investigated whether cultivable *S. symbiotica* was able to transit from the soil to the plants, and then be acquired by aphids feeding on these plants. We also analyzed the biological effects of the bacterium on host plants. In addition, we studied whether host plants can mediate horizontal transfers of cultivable *S. symbiotica* strain between aphids. Overall, our study highlights the importance to consider symbiosis in complex systems, where bacteria can circulate between different partners.

## Materials and Methods

### Insects Rearing and Bacterial Strains

The clone A06-407 of *A. fabae* used in this study was originally collected from *Chenopodium album* in St. Margrethen (Switzerland) and provided by Dr. Christophe Vorburger (University of Zurich) (Vorburger and Gouskov, 2011). The clone was found to be uninfected with any known facultative symbiont of aphids (Vorburger et al., 2009; Vorburger and Gouskov, 2011). Insects were reared on seedlings of *V. faba* at 18°C in a long-day regimen (16 h light, 8 h dark) and 65 ± 3% of humidity to ensure parthenogenetic reproduction. The cultivable CWBI-2.3^T^ *S. symbiotica* strain was used in this study. The bacterium was isolated from a natural *A. fabae* collected in Belgium (Sabri et al., 2011). Bacteria were preserved in frozen stocks at -80°C and cultured at 20°C with 863 medium (1% yeast extract, 1% casein peptone, 1% glucose) as described in (Sabri et al., 2011).

### Diagnostic PCR

Diagnostic PCR was used to check the integrity of the *A. fabae* population before experiments and to determine the presence of cultivable *S. symbiotica* in aphids and *V. faba* plants after infection. DNA from individual aphids was extracted by using a high salt-extraction method (Aljanabi and Martinez, 1997) and DNA from part of plants was extracted by using the CTAB method (Doyle, 1991). PCR primers used for *S. symbiotica* detection were 16SA1 (5′-AGAGTTTGATCMTGGCTCAG-3′) and PASScmp (5′-GCAATGTCTTATTAACACAT-3′) (Fukatsu et al., 2000). PCR reactions were performed in a final volume of 15 μl containing 1 μl of the template DNA lysate, 0.5 μM of each primer, 200-μM dNTP’s, 1X buffer and 0.625 unit of Taq DNA polymerase (Roche). The PCR reaction conditions consisted of 35 cycles at 95°C for 30 s, 55°C for 1.5 min and 72°C for 1.5 min. DNA from an infected line of the aphid *A. pisum* was used as a positive control [Tucson clones by the Nancy Moran Lab (Burke et al., 2009)]. The amplicons were then purified and sequenced in both directions (Macrogen Inc., Amsterdam). The resulting sequences were cleaned and aligned using Geneious^^®^^ v9.1.5 (Kearse et al., 2012) and compared to sequences on GenBank using BLAST to verify the identity of recovered *S. symbiotica* in aphids and plants (similarity threshold = 100%).

### Bacterial Solution and Aphid Oral Infection

A cultivable *S. symbiotica* solution was prepared and used to infect aphids and host plants. The bacteria were first grown to an early log phase in 863 medium (without antibiotic) (Sabri et al., 2011) on a gyratory shaker (160 rpm) at 20°C. When an optical density (OD) between 0.5 and 0.7 at 600 nm was reached during the logarithmic growth phase, bacteria were centrifuged. The bacterial cells were then washed with sterile PBS (Sigma), suspended in buffer and adjusted to obtain a bacterial solution with an OD of 1 at 600 nm. Oral infection was performed by feeding aphids on an artificial medium containing the bacterial solution to insure presence of the bacterium in the digestive tract of the new host (Altincicek et al., 2011). To standardized aphids, adult females of *A. fabae* were left on young *V. faba* plants during 24 h to produce nymphs. After removal of adult insects, newborn nymphs were kept on the same plants for 4 days prior to infection experiments. Third-instar aphid nymphs were then fed on an artificial diet (Cambier et al., 2001) for 24 h. One hundred μl of bacterial solution (only sterile PBS for the control treatment) was mixed with 20 ml aphid diet [corresponding to approximately 10^6^ CFU/ml of diet; as found in (Darby and Douglas, 2003; Altincicek et al., 2011; Renoz et al., 2015)]. Diagnostic PCR was carried out on some aphids to verify that these ones were infected by the bacteria.

### Environmental Acquisition Experiment

**FIGURE 1:**
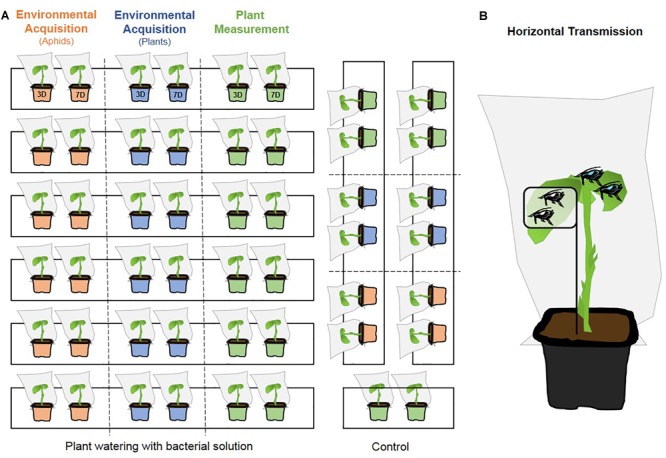
Schematic overview of environmental acquisition, plant measurement **(A)** and horizontal transmission **(B)** experiments. **(A)** Six trays were used for the bacterial infection condition and two trays for the control condition (three for the plant measurement experiment). In each tray, two plants were used to analyze the aphid bacterial acquisition (orange), two others plants were used to analyze the bacterial acquisition by plants (blue) and two others plants were used to measure plant functional traits (green). Two surveys were conducted: 3D = 3-days acquisition duration and 7D = 7-days acquisition duration. **(B)** Six to fifteen uninfected aphids (white gut) were placed inside the leaf cage on the plant and another 15 infected aphids (blue gut) were placed outside from the leaf cage, allowing the release of the bacterium on plant tissues (uninfected aphids for the control).

To study the environmental acquisition of the cultivable *S. symbiotica* by aphids, an experiment was carried out via a plant watering system with a bacterial solution. It consisted of mixing 7 ml of the solution of cultivable *S. symbiotica* or PBS (control) in one liter of 863 medium (corresponding to approximately 10^6^ CFU/ml of solution). The mixture was placed on gyratory shaker (160 rpm) at 20°C allow bacterial growth. After 1 day, each young plant (reared during 20 days at 20 ± 2°C under a long day regimen (16 h light, 8 h dark) and 65 ± 3% of humidity) was watered with 200 ml of the mixture. Six trays were used for the bacterial infection condition and two trays for the control condition (Figure 1A). In each tray, two plants (one for each survey) were used to analyze the bacterial acquisition by plants and on two others plants treated in the same way, ten aphids were placed per plant to analyze aphid bacterial acquisition (Figure 1A). After 3 and 7 days of exposure, three portions (two pieces of leaves (one on each leaf), two pieces of stems and two pieces of roots) of each plant were collected and all aphids sampled. For each survey, six replicates for the bacterial infection condition and two replicates for the control condition were performed (five to nine aphids per plant and one plant per replicate). All the material was then analyzed for the presence of *S. symbiotica* by diagnostic PCR.

### Plant Measurement

To investigate the biological effects of the cultivable *S. symbiotica* on host plants, we measure eight plant functional traits (Kraft et al., 2015): maximum shoot length (cm), maximum root length (cm), total leaf dry mass (g), total leaf area (cm^2^), total root dry mass (g), specific leaf area (SLA, cm^2^/g) defined as the ratio of total leaf area to total leaf dry mass (Wright and Westoby, 1999), specific root length (SRL, cm/g) defined as the ratio of root length to total root dry mass (Wright and Westoby, 1999) and root:shoot ratio. These traits are known to capture ecologically significant variation in leaves, roots and whole-plant function across plant species (Kraft et al., 2015). Trait measurements were performed on young plants aged 20 days, having been exposed to the plant watering system, as explained previously (Figure 1A). Whole plants were photographed using a camera (Canon EOS 450D Zoom EFS 18–55 mm). Measurements of maximum shoot length, maximum root length and total leaf area were then performed using ImageJ’s measuring function (Abramoff et al., 2004). All fresh leaves and roots of each plant were dried to a constant mass at 60°C and weighed on an analytical balance (Ohaus Explorer E12140, Switzerland). Weight measurement allowed us to determine the SLA and the SRL. The root:shoot ratio was also measured using the total root dry mass to the total leaf dry mass. After 3 and 3 days of exposure, each plant was used for the measurements, after gently washing of its root to remove the soil. For each survey, 3 replicates for the control condition and six replicates for the bacterial infection condition were performed (1 plant per replicate).

### Horizontal Transmission Test

To determine whether the cultivable *S. symbiotica* bacterium is capable of horizontal transfers between aphids via plant sap, feeding experiments on *V. faba* plants were performed. Six to fifteen uninfected third instar aphids were placed inside a leaf cage attached on a young *V. faba* plant to prevent nymphs escaping and contamination via honeydew (Figure 1B; Caspi-Fluger et al., 2012). Another fifteen third instar aphids infected by the bacterium were placed outside from the leaf cage, allowing the release of the bacterium on plant tissues (Figure 1B). A diagnostic PCR was carried out on the aphids placed in cages after 3 and 7 days as described in (Gonella et al., 2015) to test for the presence of *S. symbiotica* (4 replicates/survey). Negative controls followed the same protocol, but the uninfected aphids were exposed to uninfected aphids (three replicates/survey).

The same procedure was used to test for the presence of cultivable *S. symbiotica* in leaves placed inside leaf cages and thus to study the efficiency of cultivable *S. symbiotica* bacteria transmission from aphids to plants (three replicates/survey for the infected plants and one replicate/survey for the control).

### Histological Observations

To visualize where cultivable *S. symbiotica* strain is located in the plant, fluorescence *in situ* hybridization (FISH) was performed as previously described in (Ryuichi Koga, 2009; Caspi-Fluger et al., 2012). Visualization was performed on plants having undergone the same procedure as the watering experiment and the horizontal transmission test. Six fresh leaves and stems infested with cultivable *S. symbiotica* for 7 days by the watering experiments and twelve fresh leaves infested with the bacterium for 7 days by infected aphids of the transmission test were thinly sliced vertically with a sterile razor blade to recover part of the midrib. The samples were directly fixed in Carnoy solution at room temperature overnight. After fixation, specimens were bleached in alcoholic 6% H_2_O_2_ solution for 2 days in complete darkness and then hybridized overnight in hybridization buffer containing the fluorescent probe (10 pmol/ml): Cy3-PASSisR (5′-Cy3-CCCGACTTTATCGCTGGC-3′) targeting 16S rRNA of *S. symbiotica*. After washing, stained samples were mounted in SlowFade antifade solution (Invitrogen), and observed under a Zeiss LSM 710 confocal microscope. Negatives controls consisted of uninfected leaves and stems and no-probe staining.

### Statistical Analyses

The analysis aimed at identifying the effect of the cultivable *S. symbiotica* bacterium on the host plants. Plant functional traits were analyzed using general linear models (LM), after validation of the normal distribution, using condition (infected or uninfected plants) and survey (3 or 7 days of bacterial acquisition) as fixed factors. Statistical analyses were performed using the software R version 3.0.1 (R Development Core Team, 2014), using *GrapheR* package for graphics.

## Results

### Environmental Acquisition of Cultivable *S. symbiotica*

**FIGURE 2:**
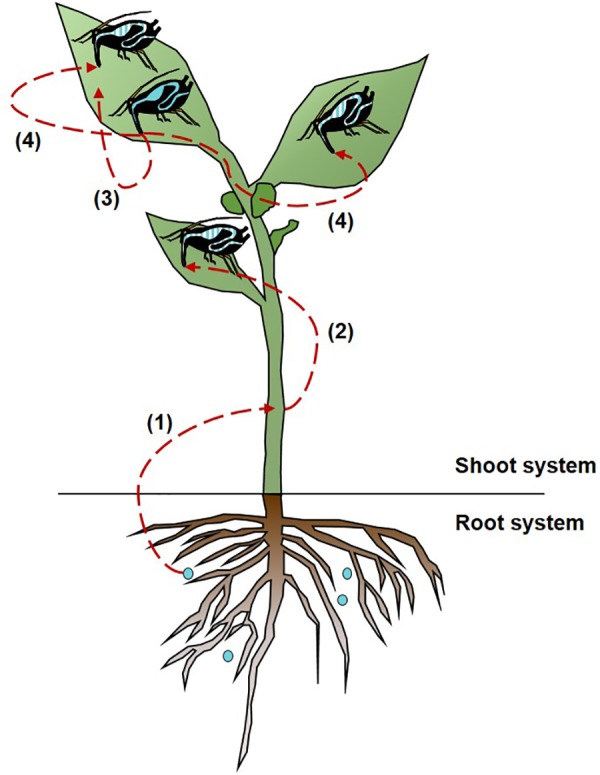
Multitrophic circulation of cultivable *Serratia symbiotica* bacteria. Cultivable *S. symbiotica* can be absorbed by plant roots **(1)**, which is a likely route for cultivable *S. symbiotica* acquisition by aphids **(2)**. The cultivable *S. symbiotica* can be transferred from aphids to plants **(3)**, and can be acquired by other aphids by horizontal transmission **(4)**. The dashed lines represent the circulation of the bacteria. The blue gut represents infected aphids with the bacteria and the blue striped gut represents infected aphids with the bacteria via the horizontal transfers.

The cultivable *S. symbiotica* strain can be transported from the soil infected with bacterial solution to *V. faba* plants [Figure 2 (1)]. After 3 days of exposure, the cultivable *S. symbiotica* was only detected in the leaves of plants (Table 1). The two pieces of leaf were infected in five out of six plants and one out of two leaf pieces was found to be infected in the other plant. After 7 days of exposure, the cultivable *S. symbiotica* was detected in both leaves and stems (Table 1). Leaves of four out of six plants were infected with the cultivable *S. symbiotica*. For one plant, two pieces of leaf were found infected and for the three other plants, only one piece of leaf was infected. In addition, stems of two out of six plants were infected. For these plants, one piece of stem was infected. Moreover, the cultivable *S. symbiotica* was not detected in the roots of plants (Table 1). For the control experiment, no part of plants was found to be infected with the bacteria (Table 1).

**Table 1:** Environmental acquisition experiment of cultivable *S. symbiotica* by plants.

Duration of *Serratia symbiotica* acquisition	Replicates	Number of infected plants by *Serratia symbiotica*					
		Number of leaf pieces tested	Number of infected leaf pieces	Number of stem pieces tested	Number of infected stem pieces	Number of root pieces tested	Number of infected root pieces
3 days	1	2	2	2	0	2	0
	2	2	**2**	2	0	2	0
	3	2	**1**	2	0	2	0
	4	2	**2**	2	0	2	0
	5	2	**2**	2	0	2	0
	6	2	**2**	2	0	2	0
	Control 1	2	0	2	0	2	0
	Control 2	2	0	2	0	2	0
7 days	1	2	0	2	0	2	0
	2	2	**2**	2	0	2	0
	3	2	**1**	2	**1**	2	0
	4	2	**1**	2	0	2	0
	5	2	**1**	2	**1**	2	0
	6	2	0	2	0	2	0
	Control 1	2	0	2	0	2	0
	Control 2	2	0	2	0	2	0

*Cultivable *S. symbiotica* frequency in uninfected plants exposed to infected (treatment) or uninfected (control) solution. Three portions of each plant were analyzed: two pieces of leaves (one on each leaf), two pieces of stems and two pieces of roots. The duration of the bacterial acquisition was 3 and 7 days.*

After 3 days, all plants had aphids infected by the cultivable *S. symbiotica* with an infection rate ranging from 22 to 75%, depending on the host plants (Table 2). After 7 days of exposure, five out of six plants had aphids infected by the bacteria with an infection rate ranging from 14 to 57%, depending on the host plants (Table 2). For the control experiment, no aphids were found infected with the cultivable *S. symbiotica* (Table 2). The cultivable *S. symbiotica* can thus be transferred from *V. faba* plants to the aphids, when bacteria were present only in the environment in direct contact with the roots [Figure 2 (2)].

**Table 2:** Environmental acquisition experiment of cultivable *S. symbiotica* by *A. fabae* aphids.

Duration of *Serratia symbiotica* acquisition	Replicates	Total number of aphids tested	Number of infected aphids by *Serratia symbiotica* [infection rate (%)]
			
3 days	1	6	**3** (50)
	2	6	**4** (66.67)
	3	8	**2** (25)
	4	8	**6** (75)
	5	9	**6** (66.67)
	6	9	**2** (22.22)
	Control 1	6	0
	Control 2	6	0
7 days	1	6	**2** (33.33)
	2	7	**4** (57.14)
	3	7	**1** (14.29)
	4	6	**3** (50)
	5	8	**2** (25)
	6	6	0
	Control 1	8	0
	Control 2	5	0

*Cultivable *S. symbiotica* frequency in uninfected aphids exposed to infected (treatment) or uninfected (control) plants. The duration of the bacterial acquisition was 3 and 7 days.*

### Effect of Cultivable *S. symbiotica* on the Host Plant

Eight plant functional traits were measured in order to investigate the biological effects of the cultivable *S. symbiotica* strain on plants. This strain had a significant effect on maximum shoot length of host plants (Tables 3, 4). Maximum shoot length was higher for plants infected with the cultivable *S. symbiotica* compared to uninfected plants (LM, *t* = 3.92, *p* = 0.0016). The cultivable bacteria had no significant effects on total leaf dry mass and total leaf area of host plants, but the interaction between condition and survey had a significant effect on total leaf dry mass (Tables 3, 4). During the 3-day surveys, total leaf dry mass was higher for infected plants compared to uninfected plants, while the results were reversed after 7-days. In addition, during the experiment, total leaf dry mass increased significantly for uninfected plants, while total leaf dry mass remained stable for infected plants (LM, *t* = -2.46, *p* = 0.028). The SLA (cm^2^/g) was significantly higher in 3-day surveys compared to 7-day surveys (LM, *t* = -5.084, *p* < 0.001), however, the cultivable bacteria did not significantly affect this trait (Tables 3, 4), meaning that host plants developed new and smaller leaves. The cultivable *S. symbiotica* strain had no significant effect on the maximum root length. Nevertheless, *S. symbiotica* had a significant effect on total root dry mass and the SRL (cm/g) of host plants (Tables 3, 4). Total root dry mass was higher for plants infected with the cultivable *S. symbiotica* compared to uninfected plants (LM, *t* = 2.38, *p* = 0.031). The SRL was lower for plants infected with the cultivable *S. symbiotica* compared to uninfected plants (LM, *t* = -2.35, *p* = 0.033), signifying that the infected plants had developed more roots horizontally and/or the roots had a larger diameter than uninfected plants. The interaction between condition and survey had a significant effect on the root:shoot ratio of the host plants (Tables 3, 4). During the 3-day surveys, the ratio was the same for infected and uninfected plants, while during the 7-day surveys, the ratio was higher for infected plants than for uninfected plants, meaning that infected plants have allocated more energy into root growth than leaves, unlike uninfected plants. In addition, the root:shoot ratio decreased significantly for uninfected plants, whereas it increased for infected plants (LM, *t* = 2.44, *p* = 0.029).

**Table 3:** Mean ± Standard Error of eight functional traits of the host plant *Vicia faba* according to infection status.

Plant functional traits	Control		Bacterial solution	
	3 days	7 days	3 days	7 days
Maximum shoot length (cm)	16.22 ± 0.58	16.18 ± 0.51	20.16 ± 0.71	19.56 ± 0.56
Maximum root length (cm)	17.67 ± 0.86	19.97 ± 1.78	19.79 ± 0.75	19.95 ± 0.57
Total leaf dry mass (g)	0.09 ± 0.01	0.14 ± 0.00	0.12 ± 0.01	0.12 ± 0.01
Total leaf area (cm^2^)	27.72 ± 4.07	30.07 ± 1.09	41.14 ± 3.58	28.31 ± 3.26
Total root dry mass (g)	0.21 ± 0.04	0.18 ± 0.02	0.25 ± 0.02	0.32 ± 0.04
SLA (cm^2^/g)	319.09 ± 10.33	208.55 ± 7.40	340.65 ± 9.23	226.04 ± 14.61
SRL (cm/g)	91.65 ± 20.13	112.34 ± 12.14	81.28 ± 6.12	67.94 ± 9.20
Root:shoot ratio	2.58 ± 0.76	1.25 ± 0.14	2.10 ± 0.17	2.60 ± 0.33

*Two treatments were used: plants uninfected with the cultivable *S. symbiotica* (control) and plants infected with the cultivable *S. symbiotica* (bacterial solution). Two surveys were carried out: 3 and 7 days after inoculation.*

**Table 4:** General linear model results for the eight traits measured.

Source of variation	d.f.	MS	*F*	*P*
**Maximum length of shoot (cm)**				
Condition	1	53.58	26.49	**<0.001**
Survey	1	0.77	0.38	0.55
Condition^∗^survey	1	0.31	0.16	0.7
**Maximum length of roots (cm)**				
Condition	1	4.37	1.2	0.29
Survey	1	3.43	0.96	0.34
Condition^∗^survey	1	4.58	1.28	0.28
**Total dry mass of leaves (g)**				
Condition	1	0.00017	0.37	0.55
Survey	1	0.002	4.14	0.061
Condition^∗^survey	1	0.0029	6.038	**0.028**
**Total leaves area (cm^2^)**				
Condition	1	136.09	1.96	0.18
Survey	1	271.52	3.91	0.067
Condition^∗^survey	1	230.41	3.98	0.066
**Total dry mass of roots (g)**				
Condition	1	0.033	5.96	**0.029**
Survey	1	0.007	1.26	0.28
Condition^∗^survey	1	0.0099	1.78	0.2
**SLA (cm^2^/g)**				
Condition	1	1525	2.15	0.16
Survey	1	57718	81.39	**<0.001**
Condition^∗^survey	1	17	0.023	0.88
**SRL (cm/g)**				
Condition	1	2999.39	6.02	**0.028**
Survey	1	17.98	0.036	0.85
Condition^∗^survey	1	1158.27	2.32	0.15
**Root: shoot ratio**				
Condition	1	0.76	1.38	0.26
Survey	1	0.057	0.1	0.75
Condition^∗^survey	1	3.3	5.96	**0.029**

*d.f., degrees of freedom; MS, mean squared; F, *F* test; and *P, p*-value.*

### Horizontal Transmission of Cultivable *S. symbiotica*

A horizontal transmission of the cultivable *S. symbiotica* was observed in aphids located on the same plant [Figure 2 (4)]. After 3 days of acquisition, infected aphids by cultivable *S. symbiotica* were found on two out of four plants, with the infection rate varying between 20 and 67%. After 7 days of acquisition, infected aphids were found on three out of four plants, with the infection rate varying between 20 and 60% (Table 5). For the control experiments, no aphids were found to be infected with the bacteria (Table 5). Bacteria were also found in leaves of host plants: two out of three leaves were infected in each survey. For the control experiment, no plant leaf was found infected with the bacteria. These results suggest that (1) aphids are able to release bacteria in plants, (2) cultivable *S. symbiotica* strain can then move within the plants, and (3) can be acquired by previously uninfected aphids.

**Table 5:** Horizontal transmission experiment of cultivable *S. symbiotica* to *A. fabae* aphids.

Duration of *Serratia symbiotica* acquisition	Replicates	Number of exposed aphids	Number of infected aphids
**3 days**	Ctrl 1	9	0
	Ctrl 2	10	0
	Ctrl 3	10	0
	1	13	**0**
	2	14	**0**
	3	10	2
	4	15	10
**7 days**	Ctrl 1	6	0
	Ctrl 2	7	0
	Ctrl 3	9	0
	1	10	**2**
	2	10	**6**
	3	11	**3**
	4	15	0

*The cultivable *S. symbiotica* frequency found in uninfected aphids exposed to infected (treatment) or uninfected (control) aphids. The duration of the acquisition was 3 and 7 days.*

### Visualization of Cultivable *S. symbiotica*

**FIGURE 3:**
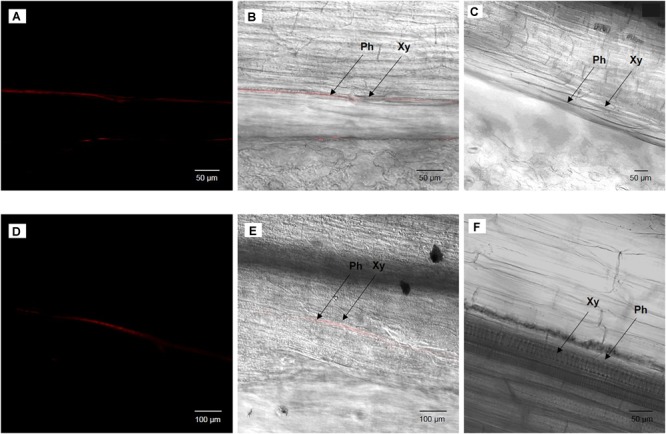
Broad bean leaf **(A,B)** or stem **(D,E)** infected by cultivable *S. symbiotica* after the watering of plants with a bacterial solution and uninfected broad bean leaf **(C)** or stem **(F)** after the watering of plants with a PBS solution (negative control), visualized by fluorescence *in situ* hybridization. **(A,D)** *S. symbiotica* channel and **(B,E)** overlay of *S. symbiotica* on bright field channels. Red Cy3 signals correspond to *S. symbiotica* in phloem. Ph: phloem and Xy: xylem.

**FIGURE 4:**
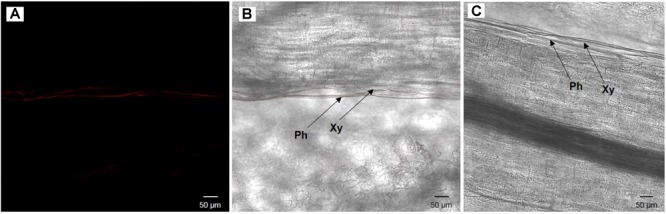
Broad bean leaf infected by the cultivable *S. symbiotica* after the presence of infected aphids **(A,B)** and uninfected broad bean leaf inoculated with uninfected aphids (negative control, **C**), visualized by fluorescence *in situ* hybridization. **(A)** *S. symbiotica* channel and **(B)** overlay of *S. symbiotica* on bright field channels. Red Cy3 signals correspond to *S. symbiotica* in phloem. Ph: phloem and Xy: xylem.

Once the presence of cultivable *S. symbiotica* in plants and aphids was determined, localization of the bacteria within plant tissues was investigated using a fluorescence *in situ* hybridization approach. For the environmental acquisition test, bacteria were found in five out of six leaves and three out of six stems of the infected plants, restricted to the phloem vessels, but not in any of the control leaves and stems (Figure 3). For the horizontal transmission test, bacteria were found in ten out of twelve leaves of the infected plants, also restricted to the phloem vessels, but not in the control leaves (Figure 4).

## Discussion

Much is known about aphids-plants (Goggin, 2007; Guerrieri and Digilio, 2008) and aphids-symbiotic bacteria (Oliver et al., 2014; Leclair et al., 2016) interactions, but understanding how aphids, plants and symbiotic bacteria interact together in nature remains largely elusive. It is established that the acquisition of environmental bacteria can have ecological and evolutionary impacts for hosts (Sugio et al., 2015). Our study clearly shows that the cultivable *S. symbiotica* having the possibility of free-living lifestyle are capable of circulating from the soil to plants, as well as between plants and aphids (Figure 2). Our study also showed direct evidence of plant-mediated horizontal transmission of the cultivable *S. symbiotica* in aphid (Figure 2). Consequently, cultivable *S. symbiotica* horizontal transmission in aphids through plants is likely an important way by which these bacteria can spread in natural systems.

The oral route is one of the most common entry sites of bacteria into animals (Bright and Bulgheresi, 2010). We showed that cultivable *S. symbiotica* can enter the plants and then can be acquired by aphids through nutrition (Figure 2). These findings suggest that the bacteria may enter and move inside plant tissues, but the molecular mechanisms whereby plants acquire this cultivable strain are unknown. Since there is a vast microbial diversity in the soil environment (Garbeva et al., 2004), specific bacteria must be absorbed selectively and efficiently to pass the defenses of the plant roots (Bendix and Lewis, 2018). Indeed, in response to infection and to avoid pathogens, plants release biologically active compounds into the rhizosphere known to act as attractants or repellents to microbial soil communities (Baetz and Martinoia, 2014). Once in the plant sap containing a low amount of microorganisms (Jing et al., 2014), the cultivable *S. symbiotica* strain must thus overcome or resist plant defenses. Our FISH results suggest that the cultivable *S. symbiotica* can be found in phloem sap, which is why we first find a bacterial infection in the leaves and then in the stem. The cultivable *S. symbiotica* strain is probably passively transported by the xylem to the leaves where the bacteria will multiply and concentrate in the phloem that the aphid feed on. Indeed, unlike xylem sap, phloem sap is rich in sugar (Douglas, 2006; Bendix and Lewis, 2018) and is therefore a suitable environment for the development of the bacterium that needs sugar for its growth (Sabri et al., 2011). This would also explain why we do not find cultivable *S. symbiotica* in the plant roots. We suggest that the bacteria are passively transported in the roots and their low concentration does not allow to detect them. The cultivable *S. symbiotica* strain can be acquired by aphids during nutrition and end up in their gut with the sap where the niche is also interesting for them. Our hypothesis is that an evolutionary leap could have occurred in some of these strains localized in the aphid gut, passing the intestinal barrier of aphids and ending up in the hemolymph, a similar environment with high sugar content, but also proteins (Moriwaki et al., 2003). To establish a stable association, a loss of virulence and the diversion of function of the bacteria in favor of the aphid are needed. Environmentally generated infections could thus represent a reservoir of new beneficial traits for hosts creating a pathway toward new symbiotic associations (Clayton et al., 2012) and opens up new questions on the nature of the interaction between cultivable *S. symbiotica* and plants.

Plant-associated *Serratia* bacteria are found in various environments and plant species. When they are associated with plants, *Serratia* genus are generally referred as to endophytes, epiphytes or rhizobacteria able to enhance the growth of their host plant through a variety of mechanisms (Berg, 2000; Selvakumar et al., 2008). So far, the bacterium *S. symbiotica* has never been described as being associated with plant species, but has been depicted as an aphid symbionts (Oliver et al., 2010). Interestingly, our findings revealed that when the cultivable *S. symbiotica* was experimentally absorbed by plants, no disease symptoms were observed in plants, whereas the bacterium may modify the functioning of the plant, mainly at the root level. The presence of bacteria can promote root growth of plants (larger root diameter and/or lateral growth). The root:shoot ratio further showed that infected plants allocated more energy to root growth compared to uninfected plants. This could be explained by the production and/or induction of phytohormones by the cultivable *S. symbiotica*. For example, certain plant-associated strains of *Serratia plymuthica* and *Serratia marcescens* are known to produce IAA (indole-3-acetic acid) that is the most common plant hormone of the auxin class, which has positive effects on plant development (Ovadis et al., 2004; Shi et al., 2010). Additionally, a recent study showed that the genome of our cultivable strain encodes proteins potentially involved in invasion and biofilm formation (Renoz et al., 2017), such as the RNA polymerase sigma factor RpoS that has a regulatory role in biofilm development in some rhizobacteria (Petersen and Tisa, 2013). We also observed that presence of the bacteria promotes shoot growth of plants at length level but fosters less shoot biomass compared to uninfected plants. This is the first time that an aphid symbiont has been shown to have positive effects on the host plant, raising questions about a possible symbiotic association between this bacterium and plants. Therefore, further experiments focusing on the lifetime of the plant should be conducted to identify long-term effects, induced mechanisms, and the ability of bacteria to persist in plants.

Plant-mediated transmission of the symbiotic bacteria *S. symbiotica* in aphids has not been reported before. Several experimental studies already showed that plant sap can mediate horizontal transfers of facultative symbionts in phloem-feeding insects, such as *Rickettsia* in the whitefly *Bemisia tabaci* (Caspi-Fluger et al., 2012) and *Cardinium* in leafhoppers (Gonella et al., 2015). Here, we showed that cultivable *S. symbiotica* can be transferred from aphids to plants and subsequently acquired by other aphids (Figure 2). Unlike virus and bacteria into insect hemolymph that can invade the salivary glands and be injected into the plant phloem (Fereres and Raccah, 2015), we supposed that the cultivable *S. symbiotica* found in the aphid gut is merely ejected via the stylet into the plant phloem and then ingested during the feeding process. This result is in line with previous phylogenetic and *trans*-infection studies that suggested possibility of horizontal transfer of aphid symbiont between both phylogenetically close and distant species but that had never been demonstrated experimentally (Darby et al., 2001; Sandström et al., 2001; Darby and Douglas, 2003; Russell et al., 2003). Nevertheless, a study recently showed a possible horizontal transmission of the aphid endosymbiont *Hamiltonella defensa* mediated by plants (Li et al., 2018). The difference is that the bacterium is endosymbiotic whereas our bacterial strain is localized in aphid gut, having a more direct contact with the environment. The study showed that the transfer was local on a single plant leaf and not in a systemic way as shown here. Moreover, uninfected aphids were only placed on an infected leaf in leaf cage (Li et al., 2018), which does not allow to determine if the transmission is carried out via plants and/or via infected honeydew as shown in (Darby and Douglas, 2003). In contrast, our study shows that the horizontal transmission is carried out via plant sap where the cultivable *S. symbiotica* circulate in the plant. Field studies showed that *S. symbiotica* bacteria are found in high occurrence in the European psyllid *Cacopsylla pyri* (Pons et al., in prep) and occur in other insects feeding on plant sap (Pons et al., in prep). These results suggest that plants could be an alternative for horizontal interspecific transmissions allowing bacteria to expand their host range and spread in natural insect lineages. In addition, these transfers could increase the possibility of gene exchange and recombination during insect-bacteria-plant interactions. Our results thus extend our understanding of transfer routes that symbiotic bacteria associated with aphids can take and provide explanations of how cultivable *S. symbiotica* is extracellularly transmitted through aphid generations (Pons et al., 2019).

Horizontal transmissions can have significant consequences for the ecology and evolution of symbiotic bacteria and their new hosts, and can be considered as a compensatory event to maintain insect symbiosis, preventing symbiont loss or replacing them in certain cases (Moran and Yun, 2015). This can be different with our model because horizontal transmission is not only an occasional transfer mode in addition to vertical transmission as observed in aphid endosymbionts (Chrostek et al., 2017) but it would be the main mode of transmission. In a recent study, we indeed showed that the cultivable *S. symbiotica* in aphid gut would be extracellularly transmitted across aphid generations (Pons et al., 2019). The host plant seems thus to be a good candidate for this type of transmission in insect feeding on plant sap. This is the case of the *Burkholderia* gut symbiont and the bean bug *Riptortus pedestris* model (Kikuchi and Fukatsu, 2014), where the beneficial bacterium is environmentally re-acquired every generation by nymphs at the gut level but the difference with our model is that *Burkholderia* is predominantly sequestered in bacterial crypts in specific regions of the insect gut, establishing a stable association. The result raises interesting questions relating to the evolution of cultivable *S. symbiotica* transmission route; specifically, will the bacteria remain strictly transmitted horizontally? Or are they likely to evolve toward vertical or mixed-mode transmission routes over the course of evolution?

Our results showed a decrease in infection rates in aphids during experiments. Indeed, during the environmental acquisition and horizontal transmission tests, the infection rate of aphids by the cultivable bacteria seemed to decline. However, for the horizontal transmission test, the number of plants with infected aphids increased. These results suggest that the ability to infect and establish associations of cultivable *S. symbiotica* with their newly acquired hosts would be low. If infection occurs randomly during feeding, it is expected that the bacterium will develop occasionally in aphids. Another study showed similar observations with the symbiont *Cardinium* in leafhoppers (Gonella et al., 2015). Successful bacterial acquisition can be due to the number of insects feeding on the host plant, the abundance of bacteria in the soil, the host plant and/or insects, but also due to plant mechanisms and environmental factors. Nevertheless, we detected some *S. symbiotica* bacteria in plants in the field where aphids infected by *S. symbiotica* were present (Pons et al., in prep) suggesting that aphids can release *S. symbiotica* into the plant and/or that the bacteria can be naturally present in the aphid environment (soil, plants). These field observations as well as our results of bacterial circulation and beneficial nature of the bacterium in plants suggest that the cultivable *S. symbiotica* would retain the ability to thrive in the environment where the potential aphid hosts prosper and that environmental generated infections could represent a reservoir for the emergence of new symbiotic associations in aphids and more widely in insects (Clayton et al., 2012; Hosokawa et al., 2016). In future studies, it will be interesting to test if the cultivable *S. symbiotica* can survive during its circulation, and if it is able to establish novel and persistent associations with aphids.

## Conclusion

In conclusion, we investigated the ability of the cultivable *S. symbiotica* strain to circulate in aphid-host plant system. Although in nature the system is much more complex, our results revealed that the cultivable *S. symbiotica* can enter the plants and induce new bacterial infections in aphids feeding on these new infected plants. We also found that the bacterium had positive effects on the host plant, mainly at the root level. Our results also demonstrated that cultivable *S. symbiotica* was horizontally transmitted between aphids across plant tissues, clearly showing that plants play a potential role in horizontal transfers of certain *S. symbiotica* strains between aphids. It is therefore possible to identify at least three routes of environmental inoculation of the cultivable *S. symbiotica* bacterium. First, infected aphids can transmit bacteria to the plant that can circulate through phloem sap and infect healthy aphids. The second possible route would start from the soil: bacteria can be naturally present and/or released from the aphid to the soil, e.g., through excrements, honeydew or cadavers. Returning to a free-living lifestyle, this can be an indirect way to colonize plants then aphids. Third, young nymphs seem able to be directly contaminated by ingesting honeydew from their infected mother just after birth (Pons et al., 2019). These results may have repercussions for our understanding of the evolutionary history of *S. symbiotica* bacteria and leads to questions about its origin. Indeed, the bacterium could be pre-adapted to develop in insects and may find an additional niche in plants and/or soil that can serve as acquisition and/or transmission routes. Alternatively, in view of our results on the beneficial nature of the cultivable *S. symbiotica* in the host plant, the bacterium could be pre-adapted to plants and/or soil and may evolve to use insects as alternative hosts (Perilla-Henao and Casteel, 2016). Our research therefore emphasizes the importance of considering symbiotic associations as a complex systems where microorganisms can circulate between different organisms in the trophic chain. Studying bacterium-aphid-plant interactions will allow a better understanding of the symbiotic bacteria lifestyle and how bacterial mutualism arise and spread in natural population.

## Author Contributions

IP designed the study, performed the laboratory work and data analysis, and wrote the manuscript. CN performed the laboratory work. FR and TH helped design the study and write the manuscript. All authors gave their final approval for publication.

## Conflict of Interest Statement

The authors declare that the research was conducted in the absence of any commercial or financial relationships that could be construed as a potential conflict of interest.
